# Gypensapogenin A-Liposomes Efficiently Ameliorates Hepatocellular Lipid Accumulation via Activation of FXR Receptor

**DOI:** 10.3390/molecules29174080

**Published:** 2024-08-28

**Authors:** Yidan Deng, Jianmei Wang, Di Wu, Lin Qin, Yuqi He, Daopeng Tan

**Affiliations:** 1 Guizhou Engineering Research Center of Industrial Key-Technology for Dendrobium Nobile, Zunyi Medical University, Zunyi 563000, China; dengyidan9320@163.com (Y.D.); 18852867110@163.com (J.W.); wudi@zmu.edu.cn (D.W.); qinlin@zmu.edu.cn (L.Q.); 2 Joint International Research Laboratory of Ethnomedicine of Ministry of Education, Zunyi Medical University, Zunyi 563000, China

**Keywords:** *Gynostemma pentaphyllum*, Gypensapogenin A, FXR, bile acids, hyperlipidemia

## Abstract

Non-alcoholic fatty liver disease (NAFLD) is one of the most common metabolic diseases encountered in clinical practice, which is characterized by the excessive accumulation of triglycerides (steatosis), and a variety of metabolic abnormalities including lipid metabolism and bile acid metabolism are closely related to NAFLD. In China, *Gynostemma pentaphyllum* is used as functional food and Chinese medicine to treat various diseases, especially NAFLD, for a long time. However, the active components that exert the main therapeutic effects and their mechanisms remain unclear. In this study, Gypensapogenin A was isolated from the total saponins of *G. pentaphyllum* and prepared as a liposomal delivery system. Gypensapogenin A liposomes could activate FXR, inhibit the expression of CYP7A1 and CYP8B1, increase the expression of CYP27A1, modulate the ratio of CA and CDCA, decrease the content of CA, and increase the content of CDCA, thus forming a virtuous cycle of activating FXR to play a role in lowering blood lipid levels.

## 1. Introduction

Non-alcoholic fatty liver disease (NAFLD) is one of the most common clinical metabolic disorders characterized by excessive accumulation of triglycerides (steatosis), which in extreme cases can lead to cirrhosis or liver cancer [1]. In the United States, cases of NAFLD are projected to increase from 83.1 million in 2015 to 100.9 million in 2030 [2]. Globally, the prevalence of NAFLD is estimated to be around 25%, with the highest prevalence in the Middle East and South America [3]. Despite the high prevalence of NAFLD and its increasing impact on world health, there are still no drugs approved for its treatment [4]. NAFLD is characterized by excessive accumulation of fatty acids [3] in liver tissues, primarily in the form of triacylglycerols (TG), and a large accumulation of liver fat is a risk factor for disease progression [5]. It is well known that bile acid metabolism has been implicated in the development of NAFLD [6,7]. Farnesoid X receptor (FXR) has been shown to regulate a number of key genes involved in glucose homeostasis, lipids and bile acid (BA) metabolism, and immune responses [8,9]. Thus, FXR activity and BA homeostasis are now recognized as key factors in the regulation of general metabolism, energy expenditure, and inflammatory processes [10,11,12]. Notably, BA levels are elevated in both plasma and liver tissue of NASH patients, suggesting an association between dysregulation of BA levels and the development of NAFLD [13,14,15]. BA metabolic genes regulated by FXR include nuclear receptor small heterodimer partner [16], cholesterol 7D-hydroxylase (CYP7A1), cholesterol 12α- hydroxylase (CYP8B1), hepatocyte nuclear factor 4α (HNF4α), and liver receptor homolog-1 (LRH-1) [17,18]. Among them, FXR induces the negative receptor SHP to inhibit the transcriptional activation of the Cyp7a1 gene by HNF4α and LRH-1 [19,20,21]. SHP negatively regulates not only genes involved in the synthesis of bile acids, but also genes involved in lipogenesis, bile acid conjugation and transport, as well as in lipogenesis and gluconeogenesis in the liver [22,23,24]. Furthermore, The FXR/SHP pathway also mediates the repressive effect of bile acids on CYP8B1 gene transcription [25,26,27]. Therefore, FXR agonists would be promising therapeutic agents for NAFLD [28].

*Gynostemma pentaphyllum* (Thunb.) Makino is widely distributed in Asia including China and other countries [29,30,31]. In China, *G. pentaphyllum* has been used as a functional food and Chinese medicines to treat various diseases, especially diabetes, with a long history dating back to the Ming Dynasty [32,33]. Previous phytochemical investigations showed that Gypenosides was the major constituent in *G. pentaphyllum* [34,35,36]. It has been reported that Gypenosides can ameliorate HFD-induced non-alcoholic steatohepatitis (NASH) through direct activation of FXR and FXR-dependent signaling pathways [37], and significantly decreasing serum total cholesterol and LDL-C levels [38]. Therefore, Gypenosides may exert hypolipidemic effects by regulating the balance of bile acid metabolism through FXR. Gypenosides usually generate secondary saponin or aglycones in the gastrointestinal tract under the action of gastric juice, intestinal microorganisms, and proteases, and uptake into the blood to play a medicinal effect. The structure-activity relationship studies showed that as the presence of sugar moieties in the saponins, aglycones were more effective than glycosides [39].

In the present study, a major triterpene aglycones, Gypensapogenin A (GpA), was isolated from the hydrolyzed products of *G. pentaphyllum* saponins. Molecular docking simulations revealed that GpA had a strong FXR affinity. However, the solubility of GpA is extremely poor. The water solubility and intestinal membrane permeability of drugs are their inherent qualities that affect oral absorption, according to the Biopharmaceutics Classifcation System (BCS) of the Food and Drug Administration (FDA) [40]. Therefore, we first encapsulated GpA into liposomes and then probed the role of Gypensapogenin A-liposomes (GpA-Lip) in ameliorating lipid accumulation through the activation of FXR to regulate BA homeostasis.

## 2. Results and Discussion

### 2.1. Isolation, Characterization and Druglikeness of Gypensapogenin A

**Figure 1:**
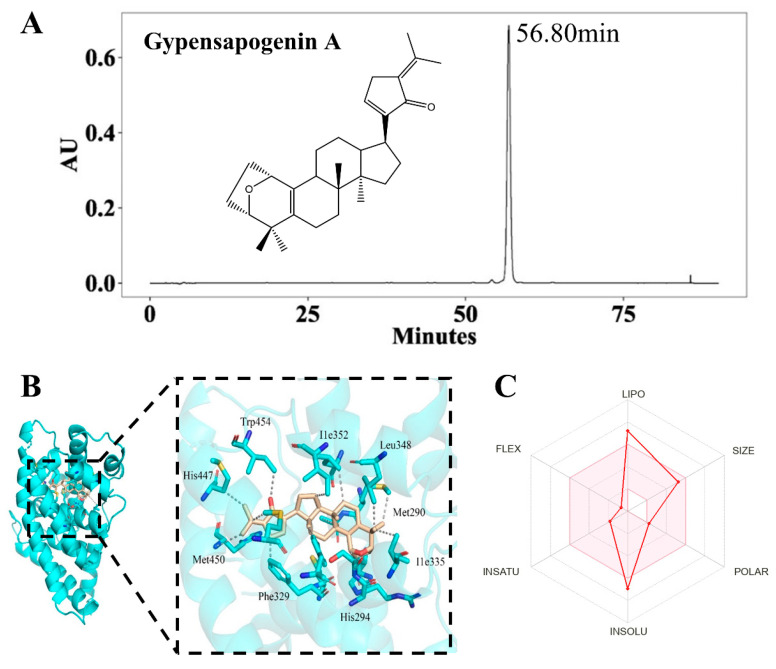
Characterization and druglikeness of GpA. (**A**) Chromatographic characterization of Gp A; (**B**) the molecular docking of GypA with FXR; Golden of Figure 1B represents the structure of GpA; Blue of Figure 1B represents the amino acids of FXR protein. Black dotted lines represent hydrogen bonds; (**C**) prediction of druglikeness properties of GypA by SwissADME. The pink area of Figure 1C represents the optimal range for each property (lipophilicity: XLOGP3 between −0.7 and +5.0, size: MW between 150 and 500 g/mol, polarity: TPSA between 20 and 130 Å^2^, insolubility: log*S* not higher than 6, insaturation: fraction of carbons in the sp^3^ hybridization not less than 0.25, and flexibility: no more than 9 rotatable bonds).

Referring to the methods in the literature [36], the total saponins of *G. pentaphyllum* were hydrolyzed and separated by column chromatography to obtain Gypensapogenin A. Its purity was 97.11%, determined by HPLC (Figure 1A), and its structure was identified by comparing its spectral data with the literature [39].

Gypensapogenin A, HR-ESI-MS: m/z 435.32 [M + H]^+^, ^1^H NMR (δ_H_, CD_3_Cl, 400 MHz): 6.95 (1H, d, *J* = 2.8 Hz, H-22), 4.34 (1H, d, *J* = 4.6 Hz, H-1), 3.79 (1H, m, H-3), 2.25 (3H, s, H-27), 1.82 (3H, s, H-26), 1.09 (3H, s, H-28), 0.83 (3H, s, H-30), 0.80 (3H, s, H-29), 0.79 (3H, s, H-18); ^13^C NMR (δ_C_, CD_3_Cl, 125 MHz): 72.9 (C-1), 24.4 (C-2), 83.0 (C-3), 38.2 (C-4), 129.1 (C-5), 19.3 (C-6), 31.6 (C-7), 37.2 (C-8), 30.8 (C-9), 131.9 (C-10), 23.0 (C-11), 30.1 (C-12), 45.4 (C-13), 47.0 (C-14), 31.3 (C-15), 23.0 (C-16), 36.6 (C-17), 21.5 (C-18), 26.1 (C-19), 152.0 (C-20), 196.1 (C-21), 145.1 (C-22), 36.0 (C-23), 128.6 (C-24),144.4 (C-25), 23.7 (C-26), 18.7 (C-27), 27.7 (C-28), 13.8 (C-29), 11.7 (C-30).

Next, molecular docking of Gypensapogenin A with FXR protein showed that Gypensapogenin A had a strong affinity for the FXR receptor (binding energy = −13.6 kcal/mol, Figure 1B). The results of the SwissADMEX analysis showed that Gypensapogenin A demonstrated good druglikeness and safety, except for its lipophilicity and insolubility exceeded the suitable physicochemical space for oral bioavailability (the colored zone in Figure 1C).

### 2.2. Parameters of Gypensapogenin A Liposomes

**Figure 2:**
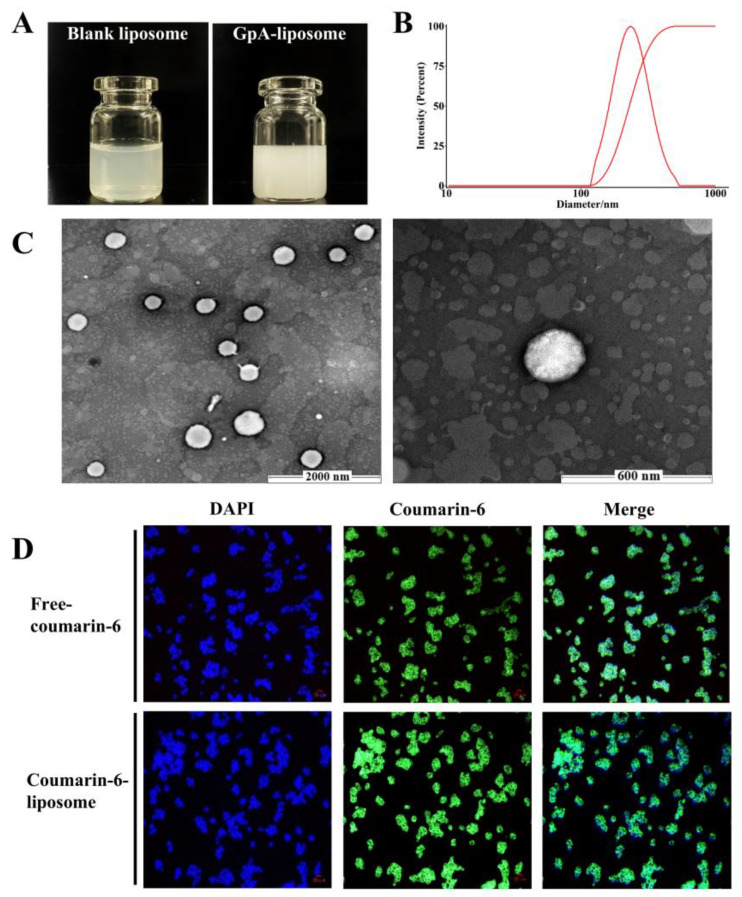
Characterization of GpA-Lip. (**A**) The samples of GpA-Lip and Blank-Lip; (**B**) particle sizes distribution of GpA-Lip; (**C**) the TEM images of GpA-Lip (scale bars = 2000 nm or 600 nm); (**D**) Cell uptake images of Coumarin-6 liposome (scale bars = 25 μm).

In order to address the bioavailability problem due to poor solubility of Gypensapogenin A, liposomes were considered as the delivery system of Gypensapogenin A. The process optimization was used to screen the Gypensapogenin A liposome, was formulated as lecithin-cholesterol-Gypensapogenin A (2:10:1) and sonicated for 15 min in PBS phosphate buffer. The encapsulation rate of the prepared liposome was 88.85 ± 0.56%; the particle size was 238.61 ± 13.07 nm, the drug loading was 8.09 ± 0.02%, the PDI was 0.106 ± 0.026, and the zeta potential was −28.91 ± 4.45 mV, which showed a uniform particle size distribution and good stability (Figure 2).

In order to examine whether the prepared liposomes could deliver the drugs into the cells, coumarin-6 was used as a fluorescent probe instead of Gypensapogenin A under the same conditions to examine the uptake of the drugs by the cells. As shown in Figure 2D, the green fluorescence excited by coumarin-6 was mainly displayed in the cytoplasm, indicating that the liposomes prepared by this process were able to deliver drugs to cells and enrich them in the cytoplasm.

### 2.3. Hypolipidemic Effects of Gypensapogenin A Liposomes

**Figure 3:**
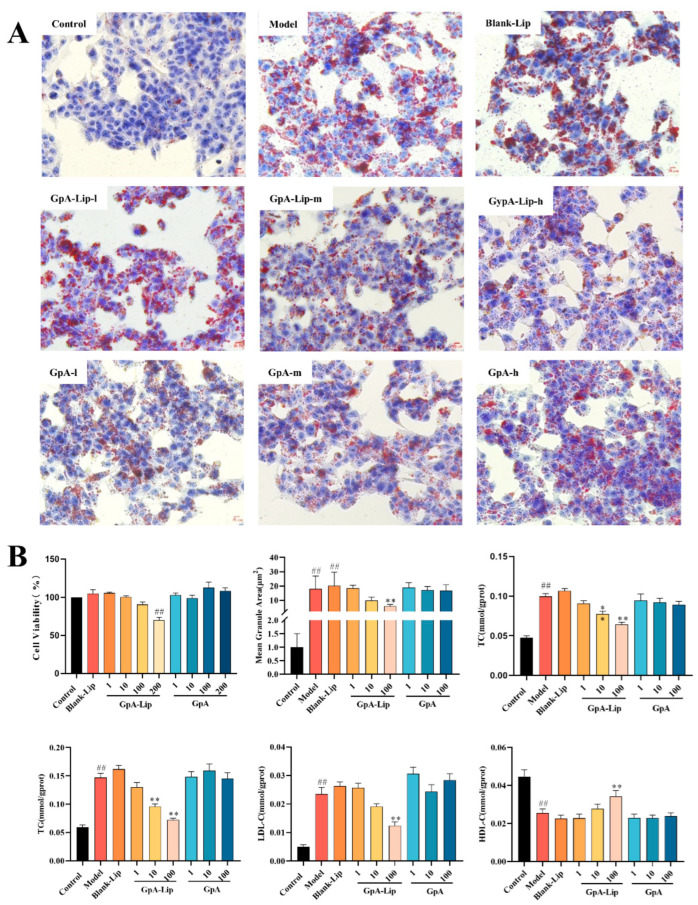
Effect of Gyp A-Lip on the amelioration of hepatocellular lipid accumulation. (**A**) Oil red O staining of the cells; (**B**) effect of GpA-Lip on biochemical indices of high-fat model cells. vs. Model (* *p* < 0.05, ** *p* < 0.01); vs. Control (## *p* < 0.01).

To evaluate the lipid-lowering activity of GpA-Lip, HepG2 cells were given FFA to establish a high-fat model, and after oil red O staining, a large accumulation of lipid droplets could be observed in the cells of the model group. After GpA-Lip intervention, the lipid droplet accumulation in the cells was reduced in a dose-dependent manner. In contrast, the phenomenon of lipid droplet accumulation in the cells of the Blank-Lip group and GpA group did not change significantly. This result suggests that GpA could exert lipid-lowering activity only when delivered to the cells via liposomes (Figure 3A).

Furthermore, biochemical analyses revealed that TC, TG, and LDL-C contents were significantly elevated while HDL-C contents were significantly reduced in the cells of the high lipid model. After GpA-Lip intervention, the levels of these indices were regressed. Similarly, there was no improvement in these parameters in the cells of the Blank-Lip group and GpA group (Figure 3B).

### 2.4. Bile Acid Analysis

**Figure 4:**
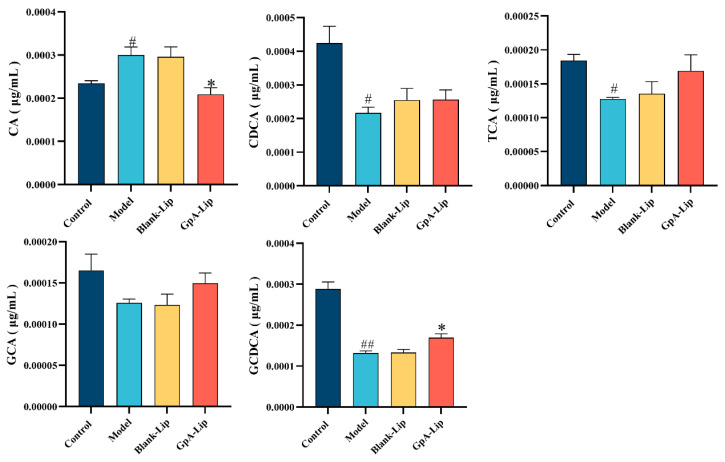
Effect of GpA-Lip on bile acids in high-fat model cells. vs. Model (* *p* < 0.05); vs. Control (# *p* < 0.05, ## *p* < 0.01).

Bile acid synthesis and excretion are the major pathways of cholesterol and lipid metabolism, which are associated with a variety of metabolic diseases, including obesity, diabetes mellitus, and nonalcoholic fatty liver disease. In human hepatocytes, cholesterol is synthesized into primary bile acids, such as CA and CDCA, mainly through the classical pathway. CA and CDCA form bound T(G)CA and T(G)CDCA by binding to glycine or taurine. According to the methods from the literature [41], five types of bile acids, including CA, CDCA, TCA, GCA, and TCDCA have been detected from HepG2 cells by using the LC-MS method. The results showed that FFA significantly elevated the levels of CA in the cells of the hyperlipidemic model, while decreasing the levels CDCA, TCA, GCA and TCDCA, compared with the normal group. After GpA-Lip intervention, CA levels in the cells were significantly reduced, while the levels of CDCA, TCA, GCA and TCDCA were somewhat restored (Figure 4).

### 2.5. Effects of Gypensapogenin A Liposomes on Bile Acids Metabolizing Enzymes

**Figure 5:**
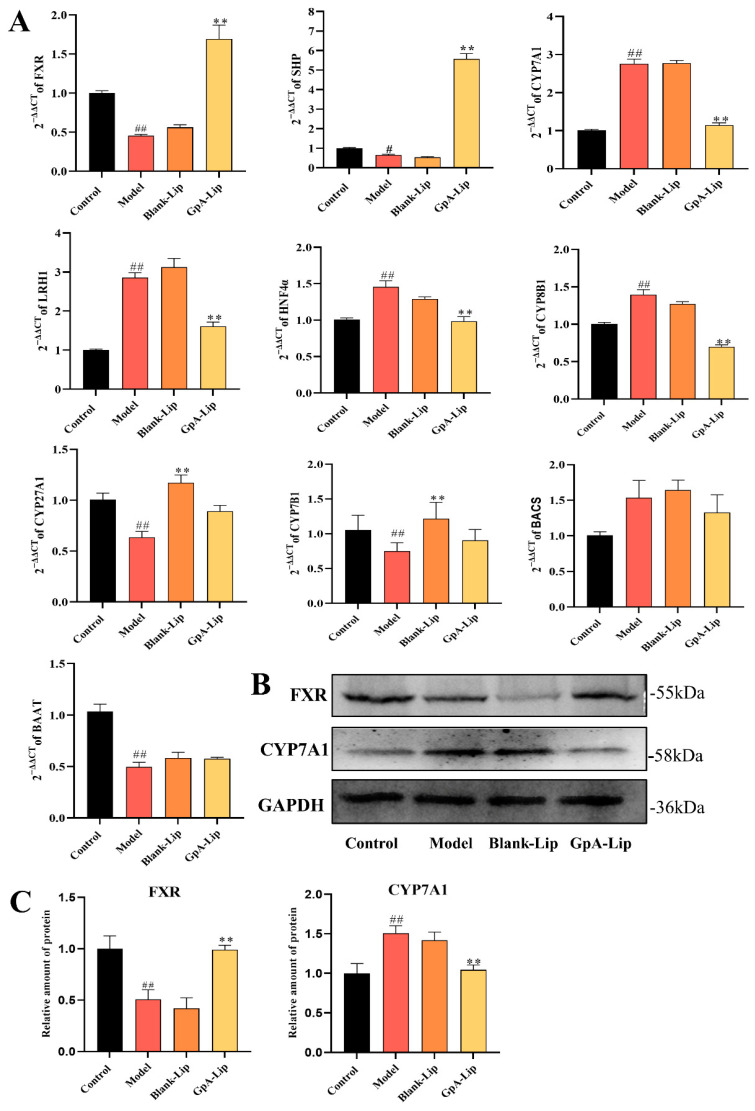
Effect of GpA-Lip on bile acid metabolizing enzymes. (**A**) Effect of GpA-Lip on the mRNA of bile acid metabolizing enzymes; (**B**) the expression of FXR, SHP and CYP7A1 proteins measured by western blotting; (**C**) effect of GpA-Lip on protein expression of FXR and CYP7A1. vs. Model (** *p* < 0.01); vs. Control (# *p* < 0.05, ## *p* < 0.01).

In human hepatocytes, cholesterol is mainly synthesized into primary bile acids, such as CA and CDCA, through the classical pathway, and further converted to T(G)CA and T(G)CDCA by BAAT and BACS. CYP7A1 is the rate-limiting enzyme of the whole pathway, which determines the amount of bile acids produced. The ratio of the two primary bile acids, CA and CDCA, is determined by CYP8B1. The expression of the CYP7A1 and CYP8B1 enzymes is regulated by the FXR pathway. In the liver, FXR induces upregulation of SHP thereby inhibiting the expression of LRH1 and HNF4α [20]. Whereas LRH1 and HNF4α are direct inducers of CYP7A1 and CYP8B1 [42], agonizing the nuclear receptor FXR therefore inhibits the expression of CYP7A1 and CYP8B1, which in turn reduces bile acid synthesis. To this end, we examined the mRNA expression levels of bile acid-related metabolic enzymes FXR, LRH1, HNF4α, CYP7A1, CYP27A1, CYP8B1, BAAT, and BACS in cells by RT-qPCR. The results showed that the mRNA expression levels of FXR and BAAT were decreased, while the mRNA expression levels of LRH1, HNF4α, CYP7A1, CYP27A1, and CYP8B1 were up-regulated, and the mRNA expression level of BACS did not change significantly in the high-fat model cells. Upon GpA-Lip intervention, the mRNA levels of all these altered metabolic enzymes were regressed (Figure 5A). This result is consistent with the changes in the levels of CA, CDCA, T(G)CA, and GCDCA detected in the cells.

In the western blotting results, it was also observed that GpA-Lip treatment reversed the decrease in FXR protein expression due to FFA. The protein expression of CYP7A1 was also reduced by the intervention of GpA-Lip (Figure 5B). These results suggest that GpA-Lip has the potential to mediate bile acids metabolism in the hepatocytes by activating FXR.

### 2.6. Mechanism Verification

**Figure 6:**
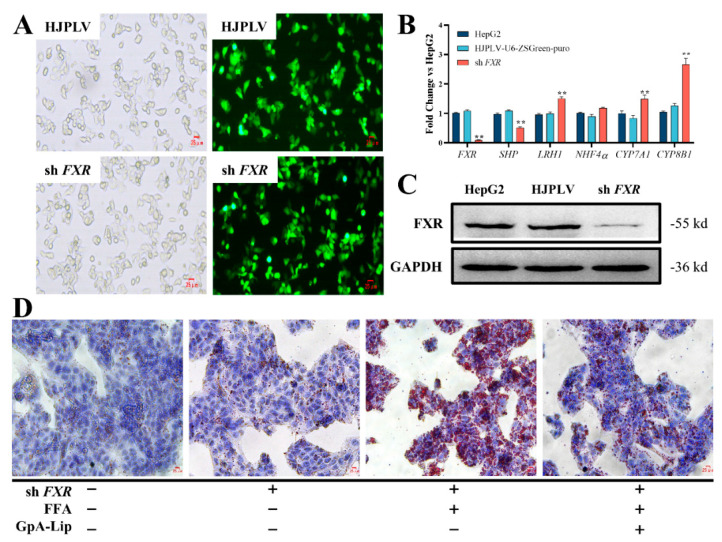

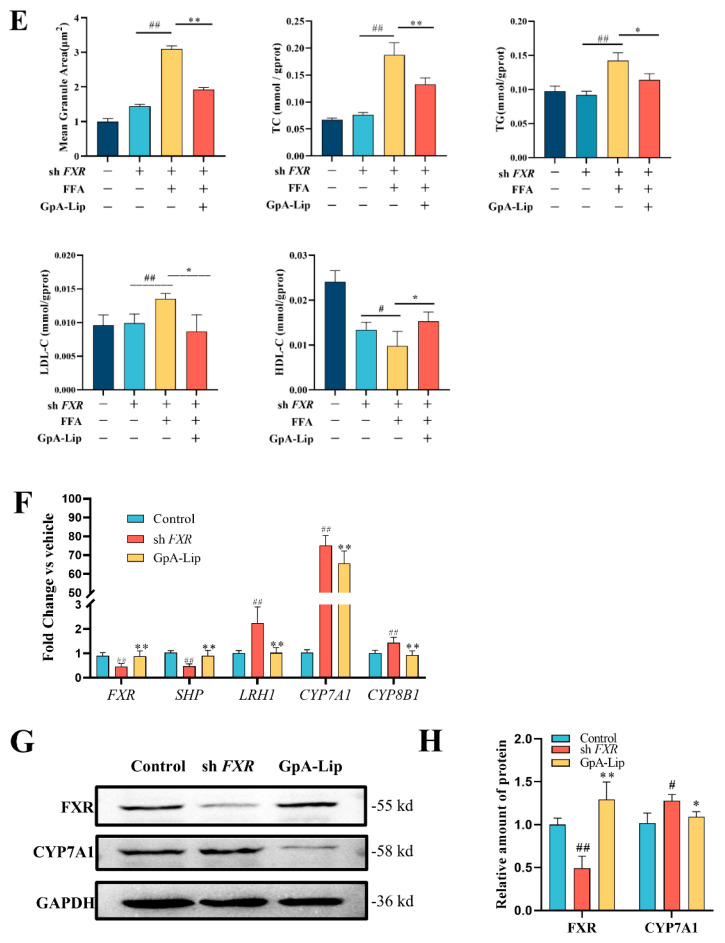
Effect of GpA-Lip on FXR knockdown cells. (**A**) FXR knockdown cell model construction; (**B**) changes in mRNA expression of key enzymes of the bile acid pathway in FXR knockdown cells; (**C**) FXR protein expression in different cells measured by western blotting; (**D**) effect of GpA-Lip on lipid accumulation in FXR knockdown cells; (**E**) effect of GpA-Lip on biochemical parameters in FXR knockdown cells; (**F**) effect of GpA-Lip on mRNA of key enzymes of the bile acid pathway in FXR knockdown cells; (**G**) the expression of FXR, SHP and CYP7A1 proteins in FXR knockdown cells measured by western blotting; (**H**) effect of GpA-Lip on protein expression of FXR and CYP7A1 in FXR knockdown cells. vs. sh FXR (* *p* < 0.05, ** *p* < 0.01); vs. Control (# *p* < 0.05, ## *p* < 0.01).

To verify whether GpA-Lip mediates the expression of bile acid metabolizing enzymes in hepatocytes by activating FXR, lentiviral vectors were used to construct FXR knockdown stable cell lines. After HepG2 cells infected by lentivirus for 48 h were screened by puromycin, HepG2-FXR-sh3 cells emitted green fluorescence when observed under a fluorescence microscope (Figure 6A). RT-qPCR analysis revealed that the expression level of FXR mRNA in the HepG2-FXR-sh3 cell line was significantly lower than that in normal HepG2 (Figure 6B). At the protein level, the results of western blotting assay were also consistent (Figure 6C). These results indicated that the FXR knockdown cell line was constructed successfully. In HepG2-FXR-sh3 cells, the expression of FXR downstream target gene SHP was significantly decreased, while the gene expression levels of LRH1, CYP7A1 and CYP8B1 were significantly upregulated (Figure 6B). Oil red O display revealed a significant increase in lipid droplet accumulation in HepG2-FXR-sh3 cells, which was significantly ameliorated by the administration of GpA-Lip (Figure 6D). Biochemical analyses showed that TC, TG, and LDL-C levels were significantly elevated and LDL-C levels were significantly reduced in FXR knockdown cells after FFA intervention, and all of these biochemical indices were significantly regressed after administration of GpA-Lip (Figure 6E). In terms of mRNA expression, the gene expression levels of FXR, LRH1, CYP7A1, and CYP8B1 in FXR-knockdown cells were significantly dialed back after treatment with GpA-Lip (Figure 6F). In terms of protein expression, western blotting results similarly showed that the protein expression of both FXR was elevated and that of CYP7A1 was decreased after treatment with GpA-Lip (Figure 6G,H).

## 3. Materials and Methods

### 3.1. Materials

The total saponins extract (>80%) of *G. pentaphyllum* was purchased from Shaanxi Zhongxin biotech Co. Ltd. (Xi’an, China), Dulbecco’s modified Eagle’s medium (DMEM, High glucose), fetal bovine serum (FBS), and 1× PBS (pH 7.4) were purchased from Heyuan Liji Biotechnology Co., Ltd. (Shanghai, China), and CCK-8 assay and was obtained from Solarbio Science & Technology Co., Ltd. (Beijing, China). Serum high-density lipoprotein cholesterol (HDL-C), LDL cholesterol (LDL-C), TG, and TC assay kits were provided by Nanjing Jiancheng Bio-Engineering Institute Co., Ltd. (Nanjing, China). Lentiviral vectors were purchased from Hejin Biotechnology Co., Ltd. (Guizhou, China). Any other analytical grade chemicals were obtained from Jinhuada Chemical Co., Ltd. (Guangzhou, China).

### 3.2. Isolation and Characterization of Triterpene Aglycones

The hydrolyzed product of total *G. pentaphyllum* saponins were obtained by dissolving in 1000 mL of methanol and then hydrolyzed by adding 500 mL of 10% HCl for 8 h under 50 °C, according to the method previously reported in the literature [36]. Next, the hydrolyzed product was loaded on a silica gel column and eluted stepwise with petroleum ether/ethyl acetate gradient (50:1–1:1) to yield Gypensapogenin A. Its structure was elucidated by ^1^H and ^13^C NMR spectral data in comparison with the literature. Its purity was calculated by area normalization under chromatographic conditions: Hypersil ODS2 (C_18_, 4.6 × 250 mm, 5.2 μm), column temperature 25 °C, injection volume 20 μL, flow rate 0.6 mL/min, scanning wavelength 203~400 nm, water (A) and acetonitrile (B) were selected as the mobile phase, and the gradient elution conditions were set as: 0–5 min, 50–60% B; 5–30 min, 60–85% B; 30–35 min, 85% B; 35–40 min, 85–90% B; 40–50 min, 90% B; 50–70 min, 93% B.

### 3.3. Molecular Docking

Gypensapogenin A was selected for molecular docking with FXR protein. The FXR protein crystal structure was obtained from the AlphaFold Protein Structure Database (https://alphafold.ebi.ac.uk, accessed on 17 December 2021) and then modified using the Autodock tools 1.5.6 software [16,43]. The 3D structure of Gypensapogenin A was generated by ChemBioDraw Ultra14.0 and converted to PDBQT coordinated by AutoDockTools. The rotatable bonds of ligands were assigned via AutoDock Tools, and the molecular docking was performed through the AutoDock Vina 1.2.2.

### 3.4. ADMET Analysis

ADMET is a comprehensive study of drug absorption, distribution, metabolism, excretion and toxicity. ADMET pharmacokinetics is a very important method in contemporary drug design and drug screening. Early ADMET evaluation of drug properties can effectively solve the problem of species differences, significantly improve the success rate of drug development, reduce the cost of drug development, reduce the occurrence of drug toxicity and side effects, and can guide the rational use of drugs in the clinic. With the development of computer technology and the accumulation of a large amount of early experimental data, the pharmacokinetic study of compounds from experimental detection to computer simulation prediction, greatly accelerating the process of drug screening. SwissADME is a commonly used ADME web analysis tool. Here, SwissADME (http://www.swissadme.ch/, accessed on 19 December 2021) was employed to predict the ADME parameters of Gypensapogenin A, including pharmacokinetic qualities, drug-like properties, medicinal chemistry friendliness etc. [44]

### 3.5. Preparation of Gypensapogenin A Liposomes

GpA-Lip was prepared using the rotary evaporation film ultrasonication method [45]. The prescribed amounts of GpA, lecithin and cholesterol were weighed in a dry flask and dissolved by ultrasonication with an appropriate amount of chloroform-methanol (3:1 *v*/*v*) mixed solvent. The organic solvent was removed by rotary evaporation under reduced pressure in a constant temperature water bath at 42 °C. After a homogeneous lipid film was formed on the wall of the bottle, the appropriate amount of phosphate-buffered saline (PBS) buffer (pH = 7.4) was added and hydrated in a rotary evaporator at 42 °C under atmospheric pressure for 2 h. Next, the suspension was dispersed through ultrasonication for 10 min under an ice bath, then filtered by a 450 nm micropore filter membrane to obtain GpA-Lip, and preserved at 4 °C.

The encapsulation efficiency of GpA-Lip was determined using a low-speed centrifugation method [46]. In this step, 1.0 mL of GpA-Lip suspension was placed in a 1.5 mL centrifuge tube and centrifuged at 1000 rpm/min for 10 min. After discarding the supernatant, 1 mL of methanol was added to dissolve it ultrasonically, and filtered through 0.22 μm filter membrane. The free GpA content in the dialysate was tested by HPLC [Chromatographic conditions: Hypersil ODS2-C_18_ column (4.6 × 250 mm, 5 μm); Column temperature was set as 30 °C; Mobile phase is acetonitrile-water solution (90:10, *v*/*v*); Flow rate was 1.0 mL/min; Detection wavelength was set as 203 nm; Injection volume was 20 μL]. The GpA-Lip encapsulation efficiency (EE%) and drug loading (DL%) were calculated according to the following Formulas (1) and (2):

EE% = (m1 − m2)/m1 × 100%(1)

DL% = (m1 − m2)/m3 × 100%(2)

where m1 is the total amount of GpA added initially, m2 is the amount of unencapsulated GpA in the dialysate, and m3 is the total amount of encapsulated GpA and pharmaceutical excipients.

The microstructures of blank liposomes (Blank-Lip) and GpA-Lip were observed by transmission electron microscopy (Micro-shot Technology Limited, Guangzhou, China), as previously described. The diameter, PDI value, and zeta potentials of the Blank-Lip and GpA-LIP were measured by a laser particle sizer (Brookhaven, MS, USA) with three replications for each sample. Stabilization was defined as the absolute value of zeta potentials above 30 mV.

### 3.6. FXR Knockdown Cells Construction

#### 3.6.1. Lentiviral Plasmid Transfection of HEK 293T Cells

When the growth density of HEK 293T cells (purchased from the Cell Bank of the Chinese Academy of Sciences, Shanghai, China) reached 80%, the constructed target plasmid, lentiviral packaging plasmid, was mixed with the basal medium, the transfection reagent was also mixed with the basal medium, and then the plasmid and the transfection reagent were co-bathed for 20 min. The mixture was dripped into the HEK 293T cells which only contained the basal medium, then replaced with a new complete medium for 6 h after the transfection, and then the supernatant of the cells was collected after 48 h and 72 h of transfection into a 15 mL centrifuge tube. After 48 h and 72 h of transfection, the cell supernatant was collected into a 15 mL centrifuge tube and filtered with 0.45 μm filter membrane to remove cell debris.

#### 3.6.2. Infection of Target Cells with Virus Solution

When the growth density of HepG2 cells reached 60–70%, the cells were infected with the virus stock solution, changed to complete medium after 9 h, and the infection rate was observed by fluorescence microscope after 48 h of infection. Finally, the target cells were screened with appropriate concentrations of antibiotics, and the screened target cells could be used to detect the expression level of genes after FXR interference by fluorescence quantitative PCR and western blotting to determine the success of the construction of FXR knockdown cells.

### 3.7. Cell Culture

HepG2 cells (purchased from the Cell Bank of the Chinese Academy of Sciences, Shanghai, China) were cultured in Dulbecco’s modified Eagle’s medium containing 10% fetal bovine serum and 1% penicillin-streptomycin in a humidified incubator at 37 °C with 5% CO_2_. For reference [47], HepG2 cells were incubated with 1 mM FFA (OA/PA, 2:1) for 24 h and then treated for 24 h by adding medium containing GpA-Lip solution or GpA solution with 5% FFA-free bovine serum albumin (BSA).

HepG2 cells in logarithmic growth phase (5 × 10^4^ cells per well) were inoculated into 96-well culture plates for 24 h. After replacing the medium with fresh medium, blank liposomes were used as controls, and the cells were further treated with GpA-Lip (1, 10, 100, and 200 μg/mL) or GpA (1, 10, 100, and 200 μg/mL) diluted in the culture medium for 24 h. Cell viability assay was performed according to the instructions of CCK-8 reagent. The absorbance of the plate was read at 450 nm.

### 3.8. Oil Red O Staining

HepG2 cells in good condition were inoculated into 6-well plates with 2 × 10^5^ cells per well and cultured overnight at 37 °C with 5% CO_2_ for 24 h. HepG2 cells in DMEM medium were used as a blank control group, and HepG2 cells inoculated into DMEM medium containing 1 mM FFAs were used as a model group. The cells were treated with different concentrations (1, 10, 100 μg/mL) of GpA-Lip under the conditions of the model medium, and were incubated for 24 h. After incubation, the cells were stained with oil red O according to the manufacturer’s protocol. Cells from each group were washed twice with PBS and fixed with 4% paraformaldehyde for 20 min at room temperature. After being rinsed with PBS and 60% isopropanol solution for 20 s, HepG2 cells were stained with 60% filtered oil red O stain (Solarbio, China) for 20 min at room temperature and protected from light. The oil red O stain was discarded and rinsed with distilled water 5 times. The nuclei were restained with Mayer’s hematoxylin solution for 2 min and washed 5 times, and finally the stained lipid droplets were observed with an inverted microscope.

### 3.9. Bile Acids Analysis

Bile acids analysis of HepG2 cells was processed as described previously [41], the bile acids and internal standard (IS) mixed reference solution was prepared with final concentrations of 1000 ng/mL TCA, TCDCA, TDCA, CDCA, LCA, 7-Keto LCA, 12-Keto LCA, αMCA, 3βHDCA, ACA, GHCA-3S, IDCA, and 5000 ng/mL CDCA-24G, TCDCA-3G, and GLCA-3S. HepG2 cells (approximately 1 × 10^7^ cells/mL per sample) were homogenized for 2 min with two magnetic beads in 300 μL tri-distilled water on ice. For the next step, 200 μL HepG2 cells homogenate was mixed with 32 μL IS (5 μg/mL), and 568 μL acetonitrile and centrifuged 15 min at 14,208× *g* at 4 °C. The supernatant (600 μL) was removed and evaporated by nitrogen gas blow, and then dissolved in 100 μL 50% methanol for LC-MS/MS analysis.

The present bile acids analysis was performed by API 4000^TM^ triple quadrupole tandem mass spectrometer system (AB ScienX Analytical Instrument Trading Co., Ltd., Shanghai, China). The XBridge BEH C18 chromatographic column (150 mm × 4.6 mm, 3.5 μm) was employed for the separation. For the mobile phase, 0.1% formic acid water (A) and acetonitrile (B) were selected, and the gradient elution conditions was set as: 0–0.5 min, 5% B; 0.5–1 min, 5–27% B; 1–2 min, 27–30% B; 2–10 min, 30–35% B; 10–15 min, 35–40% B; 15–20 min, 40% B; 20–24 min, 40–55% B; 24–27 min, 55% B; 27–34 min, 55–65% B; 34–40 min, 65–95% B. Flow rate: 0.5 mL/min, injection volume: 2 μL, column temperature: 30 °C.

Positive ion multiple reaction detection mode (MRM) mass spectrometry data was acquired using an electrospray ionization source (ESI) mass spectrometer. ESI-MS/MS parameters were set as: ion spray voltage 4500 V, curtain gas 25 psi, ion source gas1 40 psi, ion source gas2 45 psi, temperature 450 °C, and collision gas 8, respectively.

### 3.10. Biochemical Analysis

After drug treatment, the primary medium was discarded and cells were washed three times with PBS. Cells were lysed with Triton-X lysis buffer for 30 min and then centrifuged at 12,000× *g* for 10 min at 4 °C. The supernatant was then collected and the levels of TG, TC, LDL-C, and HDL-C in each well were measured according to the manufacturer’s instructions. Protein levels in each well were measured with a BCA assay kit according to the manufacturer’s instructions.

### 3.11. RT-qPCR Analysis

Total RNA was extracted from HepG2 cells using the TransZol Up reagent according to the manufacturer’s instructions. Using a reverse transcription kit, the RNA was converted to complementary DNA (cDNA) using the SYBR Green QPCR Master Mix, and a real-time quantitative polymerase chain reaction (RT-qPCR) was performed. The primer sequences are shown in Table 1.

### 3.12. Western Blotting Analysis

Cells were collected, fully lysed with RIPA on ice, supernatants were taken by centrifugation (12,000 r/min, 10 min), BCA proteins were quantified, protein samples containing 1× SDS-PAGE uploading buffer were made, 10% SDS-PAGE gel electrophoresis was performed, electrotransferred to PVDF membranes, and subsequently incubated with the corresponding antibodies, where the strips were analyzed by ECL exposure. Quantification was performed using ImageJ software 1.54f (Stuttgart, Germany).

### 3.13. Statistical Analysis

The results were presented as the mean ± SEM. Data collected were analyzed by using GraphPad prism (version 10.0, San Diego, CA, USA). Comparisons between groups were performed by one-way ANOVA followed by post hoc tests. *p* < 0.05 was considered statistically significant. All experiments were performed in triplicate.

## 4. Conclusions

**Figure 7:**
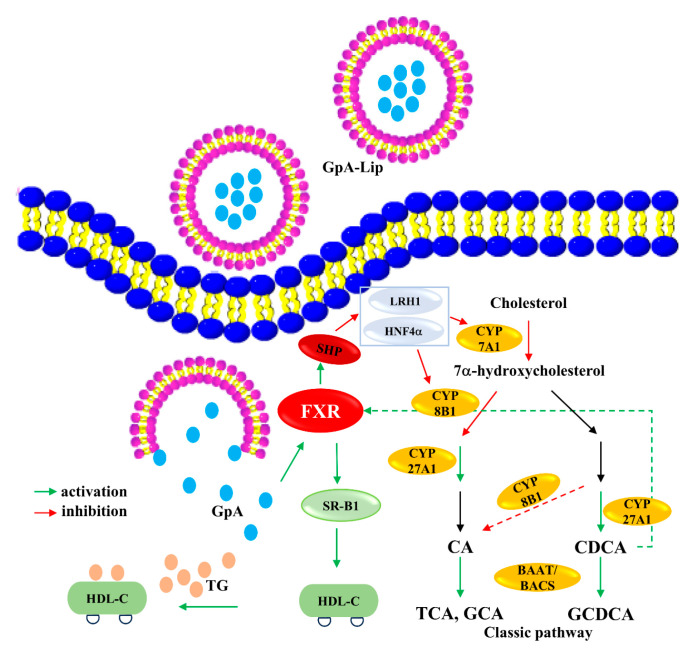
The potential mechanisms of GpA-Lip ameliorated hepatocellular lipid accumulation.

NAFLD, caused by dysregulation of hepatic lipid metabolism, has become the most common liver disease worldwide. Currently, treatment options for NAFLD/NASH patients are very limited, although FXR agonists have shown promising applications. In addition to the already marketed obeticholic acid, several FXR agonists, including Cilofexor (GS9674, Gilead, Forster City, CA, USA), are in clinical trials. FXR activation has been shown to be associated with a variety of clinical changes such as decreased liver and plasma TG levels, reduced inflammation, and increased insulin sensitivity [28,48]. Activation of FXR upregulates scavenger receptor, class B type 1 (SR-B1) expression accelerating HDL-C-promoting TG clearance [49]. In the present study, we found that Gypensapogenin A can activate FXR, inhibit the expression of CYP7A1 and CYP8B1, and increase the expression of CYP27A1, thus regulating the ratio of primary bile acids CA and CDCA, lowering the content of CA and increasing the content of CDCA. CDCA is the most effective endogenous activator of FXR, thus forming a virtuous cycle of activating FXR to lower the lipid level (Figure 7). Poor water solubility is the main problem limiting the efficacy of Gypensapogenin A. However, the lipid-lowering effect of Gypensapogenin A can be exerted by preparing it into liposomes and delivering it into hepatocytes. The results of the present study provide a preliminary explanation of the main active components and their mechanisms by which *G. pentaphyllum* exerts its hypolipidemic effects in the clinical setting. However, the efficacy and safety of GpA-Lip need to be further validated in high-fat model animals.

## Figures and Tables

**Table 1:** Primer sequences used for RT-qPCR.

Gene	Forward Primer (5′ to 3′)	Reverse Primer (5′ to 3′)
*FXR*	GCGACAAGTGACCTCGACA	TGGTTGCCATTTCCGTCAAAA
*CYP7A1*	CATGCTGTTGTCTATGGCTTATTC	ACAGCCCAGGTATGGAATTAATC
*CYP8B1*	GAGGACAGCCTCTTTCGCTT	TGTAGCCGAACAAGCTCAGG
*LRH1*	AGCAGGCTAACCGAAGCAAG	TGGAATAGTCCACTTGTTGCC
*HNF4a*	AAGAGGAACCAGTGCCGCTACT	GCTTGACCTTCGAGTGCTGATCC
*CYP27A1*	GCAACGGAGCTTAGAGGAGA	CAGGTTCACGTGCATCTGAG
*CYP7B1*	AGTGCGTGACGAAATTGACC	CAAGTCTCCCTTTCGCACAC
*BACS*	CTGAGAACATCCGCTGCTTC	ATAGATGAAGAGGGCAGGGC
*BAAT*	CCCCGCAAACCAGAAGTAAC	GAAGGGGCTGATGGATCTGA
*GAPDH*	CAGCCTCAAGATCATCAGCA	ATGATGTTCTGGAGAGCCC

## Data Availability

The authors confirm that the data supporting the findings of this study are available within the article.

## Abbreviations

BAAT	Bile Acid-CoA: Amino Acid N-Acyltransferase
BACS	Bile Acid CoA Synthase
BCA	Bicinchoninic Acid
CA	Cholic Acid
CCK-8	Cell Counting Kit-8
CDCA	Chenodeoxycholic Acid
CYP7A1	Cytochrome P450 7A1
CYP7B1	Cytochrome P450 7B1
CYP8B1	Cytochrome P450 8B1
CYP27A1	Cytochrome P450 27A1
FFA	Free Fatty Acids
FXR	Farnesoid X Receptor
GCA	Glycocholic Acid
GCDCA	Glycochenodeoxycholic Acid
GpA	Gypensapogenin
GpA-Lip	Gypensapogenin A-liposomes
HDL-C	High-Density Lipoprotein Cholesterol
HNF4α	Hepatocyte Nuclear Factor 4 Alpha
LDL-C	Low-Density Lipoprotein Cholesterol
LRH-1	Liver Receptor Homolog-1
NMR	Nuclear Magnetic Resonance
TC	Total Cholesterol
TCA	Taurocholate Acid
TG	Triglyceride
